# A Parametric Model of the LARCODEMS Heavy Media Separator by Means of Multivariate Adaptive Regression Splines

**DOI:** 10.3390/ma10070729

**Published:** 2017-06-30

**Authors:** Mario Menéndez Álvarez, Héctor Muñiz Sierra, Fernando Sánchez Lasheras, Francisco Javier de Cos Juez

**Affiliations:** 1 Department of Exploration and Mining, Universidad de Oviedo, EIMEMO, c/ Independencia 13, 33004 Oviedo, Spain; mariom@uniovi.es (M.M.Á.); hmunizsierra@gmail.com (H.M.S.); fjcos@uniovi.es (F.J.d.C.J.); 2 Department of Construction and Manufacturing Engineering, Universidad de Oviedo, Campus de Viesques, 33204 Gijón, Spain

**Keywords:** heavy media separation, density separations, multivariate adaptive regression splines (MARS), LARCODEMS

## Abstract

Modeling of a cylindrical heavy media separator has been conducted in order to predict its optimum operating parameters. As far as it is known by the authors, this is the first application in the literature. The aim of the present research is to predict the separation efficiency based on the adjustment of the device’s dimensions and media flow rates. A variety of heavy media separators exist that are extensively used to separate particles by density. There is a growing importance in their application in the recycling sector. The cylindrical variety is reported to be the most suited for processing a large range of particle sizes, but optimizing its operating parameters remains to be documented. The multivariate adaptive regression splines methodology has been applied in order to predict the separation efficiencies using, as inputs, the device dimension and media flow rate variables. The results obtained show that it is possible to predict the device separation efficiency according to laboratory experiments performed and, therefore, forecast results obtainable with different operating conditions.

## 1. Introduction

Density separation is generally considered to be the most cost effective industrial material density separation process. It is comparatively simple when compared with other techniques, and easily automated. A large variety of density separation processes and devices exist and experience in the mineral processing sector has shown that the type of density separation method or device used must be selected according to the proximity in the density of the particles to be separated and their size [1].

Density media separation (DMS) is generally considered to be the most precise type of density separation technology [2]. It is extensively used to separate materials of different densities where those of a density lower than that of the separation media float, and those greater sink, in the media. It is used for the recovery of a number of types of minerals, non-ferrous metals and plastics. In the plastic recycling sector, it is a standard method for preparing many plastic wastes for subsequent separation methods or to produce a final marketable product. It is possible to process large volumes of materials with a much wider range of particle sizes than other density separation devices. However, the efficiency obtained is susceptible to: yield stress and viscosity effects due to ultrafine suspended media particles; high solids content of the separation media; and the abundance of particles with densities very close to that of the separation media.

DMS cyclones are well established as the most precise type of density separation devices and as having very high throughputs. DMS cyclones traditionally have a cono-cylindrical form and use centrifugal forces developed in the cyclones to accelerate the float/sink separation of particles by density. Separation with this type of cyclone is accomplished by tangentially feeding the materials to separate along with the media into the cyclone, developing centrifugal forces which facilitate the rapid settling of dense particles and the floating of light particles. This is especially important for separating fine particles or those with densities approaching that of the separation media. DMS cyclones have become well established for the separation of particles from 0.5 to 50 mm. Pure density separation products are not usually obtained with this type of separator as the separation media carrying the particles drag or short-circuit some of the low-density particles to the high-density (sink) product being obtained. The degree of this short-circuiting tends to be a function of the volume of media reporting with the sink product.

There is a significant potential for increasing the volumes and range of particles sizes of waste plastics separated based on the centrifugal forces developed within DMS cyclones, as outlined by [3]. These cyclones are the most versatile, but most complicated among the density separation devices due to their operational parameters. There are a number of parameters that affect the separation quality obtained, including: separation media viscosity; media part size distribution; media flow rate and separation media pressure; cyclone diameter, length, feed and exit port sizes; and the particle size distribution of materials to process, as well as the proportion of these particles having densities in proximity to that of the separation media density.

The large coal density media separator (LARCODEMS) originally developed by British Coal is a version of the cylindrical version of a DMS cyclone. It is being used effectively for processing 0.5–120 mm particles. This device is quite advantageous compared to the cono-cylindrical type of DMS in that it is suitable for processing a wide range of particle sizes simultaneously and that the material to be processed can be fed dry into the vortex of the cyclone. This negates the need to having to pass through the separation media pumping circuit. In the case of this cylindrical type cyclone, when particles are fed into the vortex of the cyclone, the high-density particles must sink through the separation media to exit as a product without the possibility of short-circuiting occurring. Subsequently, this product fraction contains a minimum, or no, low-density particles. However, it can also be operated where the particles are fed with the separation media, as is done with the cono-cylindrical type. The separation media flow paths in the cono-cylindrical and cylindrical type cyclones are quite distinct and result in distinct types of concentrations. To date the only publication known to the authors that treats the operating parameters of the cylindrical type of DMS cyclone is that of Venkoba Rao et al. [4]. Unfortunately, no conclusions can be derived from this article as to the means for optimization of separations with this type of device. There is no public domain information as to the operating parameters of cylindrical DMS cyclones. A more theoretical understanding of the process and its design is necessary for the optimization their application. DMS cyclone separation tests have been conducted with irregularly-shaped particles with a wide range of particle sizes using a 110 mm diameter demonstration model of LARCODEMS. Results highlight that the highest-density product fraction produced was virtually pure [5].

The reported quality of density separations obtained with the LARCODEMS relative to cylindrical cyclone diameter (300 mm to 1200 mm) appears to remain consistent or to improve as the cyclone diameter is increased [6,7]. This characteristic is contrary to that of hydrocyclone particle size classification where the separation efficiency of fine particles increases with the reduction in cyclone diameter due to the higher centrifugal forces present. This improvement is probably due to the increase in particle flow path and separation residence time with the cyclone diameter. Since longer, flaky, or finer particles tend to separate more slowly, the increase in particle residence time facilitates separation of these particles. Lengthening the separation cylinder increases the separation media flow path and, as such, the particle residence time. However, the extent to which the length of the cyclone may be realistically increased remains to be determined.

### The LARCODEMS

Originally designed for treating coal, but also used for processing iron ore and plastics [6], the LARCODEMS is currently manufactured in six versions (300, 500, 850, 1000, and 1200 mm diameters) with recommended processing capacities and maximum particle sizes varying in proportion with the diameter of the apparatus. Even the smallest version has an excessive capacity for laboratory scale tests. The smallest version (300 mm diameter) has a calculated capacity to treat 6 tons/hour of plastics.

**Figure 1:**
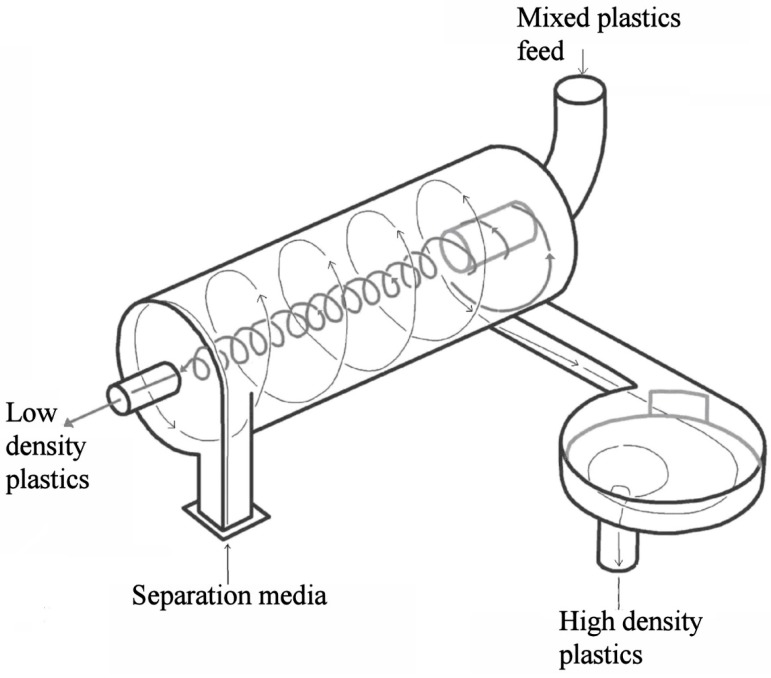
Schematic diagram of the large coal density media separator (LARCODEMS) (courtesy of JMC Engineering Ltd.).

The device consists of a cylindrical separating chamber (Figure 1) inclined at 30° from the horizontal, with the separating media pumped tangentially into the lower end. The media circulates up the interior surface of the separation cylinder until it reaches the top end where part of the media returns down in counter current along the surface of the media going up the cylinder. A vortex is formed (VF) with a central air core when a sufficient volume of separation media is pumped into the separation cylinder. The part of the media not returning down the cylinder exits tangentially through a sink port. Material to be treated is fed into the top end of the vortex so that dense fragments must settle through the descending separating media so as to reach the ascending media circulating around the inner circumference of the cylinder and exit through the sink port. Low-density particles float down the vortex to exit through a central float port (FP) at the bottom end of the cylinder. A type of siphon known as a vortex extractor can be connected (VE) to the sink port (SP). The VE provides stability to the vortex when there are fluctuations in the media flow rate into the separation cylinder and permits its operation with a significantly greater range of media input flow rates. It has been observed that as the diaphragm area (DA) is increased so does the diameter of the top end of the vortex. The diameters and, thus, the areas of the SP and FP regulate the media flow rate required to form the vortex and the rates of sink and float flow (FF and SF) exiting their corresponding ports. If one port diameter is maintained constant and the other reduced, the rate of the media flow rate of the port of constant diameter increases.

To date virtually no research has been published as to the optimization of separation efficiencies based on the design of the LARCODEMS or other any cylindrical cyclone separators. The two publications that are of any relevance in this regard are those of Chiné and Ferrtara [8] and Yang and Wang [9]. Unfortunately, only the second study has conducted any investigation as to the effect of cylindrical cyclone design parameters on the efficiencies of the separations obtained.

The aim of the present research consists of creating mathematical models for the operation of the LARCODEMS in the established mode of feeding material to be separated into the vortex and an alternative procedure of feeding this material along with the separation media. The models obtained take into account the dimensional characteristics of the device and the volume flow of dense and light materials that are introduced. The utility of this type of model is two-fold; on the one hand, they predict the efficacy of the device with different dimensions and operation conditions and, on the other hand, allow one to propose changes and design improvements having a forecast of how the device will behave with such changes.

## 2. Materials and Methods

### 2.1. Description of LARCODEMS Test Procedure

The 110 mm diameter laboratory version of LARCODEMS was designed to be operated with a 12, 21.4, 32.9, and 45 cm long separation cylinder lengths (CL). Three scaled versions (52, 72, and 172 mm diameter) of the 45 cm long 110 mm LARCODEMS were built for inclusion in these tests. Since these versions were scaled to the same relative dimensions of the 110 mm model, the relative cylinder, SP, FP, and diaphragm areas (DA) are constant. 

Fragments and pellets of 1.5 to 2.8 mm of different types of plastics with a wide range of densities were selected based on their differences in densities and differences in visual appearances for the separation tests. Densities of these were controlled to 0.002 g/cc by weight to volume ratios determined in methyl alcohol (to avoid air bubbles adhering to the plastic particles). The types were selected such that their densities (e.g., 0.940, 0.955, 0.962, 1.002, 1.034, 1.043, 1.143, and 1.160) were both greater and less than that of the separation media (water). Approximately 2000 particles for each density were combined and fed into the vortex of the LARCODEMS. The processing within the device was accomplished within some 20 seconds. The SP and FP products obtained for a given test were dried, sorted manually by plastic type, and each fraction weighed. This setup of the test procedure required some 15 hours per sample to complete. The efficiency of the separation obtained was determined as the difference in the percentage of recovery of particles denser than 1.0 g/cc in the sink product and the percentage of recovery of particles with a density <1.0 g/cc in the sink product.

The following are the variables that were employed as input data for the models of the LARCODEMS efficiency feeding either into the vortex or with media are:

(A) Separation tests with the plastic particles being fed into the vortex were conducted with:

- Twelve, 21.4, 32.9 and 45 cm long separation cylinder versions with and without a vortex extractor of the 110 mm LARCODEMS.
- Fifty-two, 72, and 172 mm diameter versions of the scaled 45 cm long, 110 mm LARCODEMS.
- Variations in diameter of the feed DA of the 110 mm LARCODEMS.
- Variations in FPA of the 110 mm LARCODEMS.
- Variations in SPA of the 52, 72, 110, and 172 mm LARCODEMS.
- Three variations in total media feed flow rate for all of the above variables when separations of plastic particles that had received a treatment (TM) to reduce hydrophobic effects were conducted without a VE.
- Five variations in total media feed flow rate for all of the above variables when separations were conducted without a VE.

(B) Separation tests with the plastic particles being fed with the separation media (FPM) were conducted with:

- The 32.9 cm-long and the 45 cm-long separation cylinder versions with and without a vortex extractor of the 110 mm LARCODEMS.
- Variations in diameter of the float port of the 110 mm LARCODEMS.
- Three variations in total media feed flow rate for all of the above variables when separations of plastic particles that had received a treatment (TM) to reduce hydrophobic effects were conducted without a vortex extractor.
- Five variations in total media feed flow rate for all of the above variables when separations were conducted without a vortex extractor.

Since the four different versions of the LARCODEMS used in this investigation were scaled to almost identical proportions, the volumes of media flow are a function of the area of the exit ports and the feed diaphragm relative to the area of the separation cylinder.

### 2.2. The Multivariate Adaptive Regression Splines

Multivariate adaptive regression splines (MARS) are techniques in the family of multivariate nonparametric regression, based in the adjustment of its parameters to the data to be modelled. These types of models were introduced by Friedman in 1991 [10]. In other words, MARS is a multivariate method able to generate models based on several input variables. The use of MARS in the present research is due to its ability to model nonlinearities and interaction between parameters. At the beginning of the research, the use of linear regression models was checked without satisfactory results. The main advantage of MARS is that it is able to effectively model relationships and patterns that are not able for other regression methods. MARS are based on measures of explanatory variables $\vec{X}$ on sizes nxp for predicting values of the continuous dependent variable $\vec{y}$, of size nx1. The MARS model can be represented as:

(1) $$\vec{y}=f(\vec{X})+\vec{e}$$

where $\vec{e}$ is the error vector of dimension nx1. With classification and regression trees (CART) models as the base [11], MARS can be considered a generalization with the capacity of overcoming some of the limitations of CART, as it does not require any information a priori relating the relationships between dependent and independent variables. CART is a statistical method for multivariate analysis that creates a decision tree which strives to correctly classify the members of a population. The MARS regression model is constructed by piecewise polynomials, also called splines, which have smooth connections. This is performed through fitting basis functions to distinct intervals of the independent variables. The polynomials joining points are named as t, and are known as knots, nodes, or breakdown points. The splines for MARS are polynomials, concretely two-sided truncated power functions, which can be expressed as follows [12,13]:

(2) $${\left[+(x-t)\right]}_{+}^{q}={\{}_{\begin{array}{cc} 0 & x<t \end{array}}^{\begin{array}{cc} {\left(t-x\right)}^{q} & x\geq t \end{array}}$$

Considering M basis functions, a MARS model can be expressed with the next expression of the estimation of the dependent variable [14]:

(3) $$\hat{\vec{y}}={\hat{f}}_{M}\left(\vec{x}\right)=c_{0}+\sum_{m=1}^{M}c_{m}B_{m}\left(\vec{x}\right)$$

where $c_{0}$ is a constant, $c_{m}$ the coefficients of the $m^{th}$, and its correspondent $m^{th}$ basis function is $B_{m}(\vec{x})$.

Then, MARS models use, as required inputs, the model and the knot positions for each individual variable to be optimized. For a dataset $\vec{X}$ containing n objects and p explanatory variables, then, the number of basis functions would be N=nxp pairs of spline basis functions, given by the above equations Equation (2), with knot locations $x_{ij}$, with i=1,2...,n and j=1,2...,p.

To reach the expression of the model that MARS provides, a selection of basis functions in consecutive pairs is necessary. The selection of the basis functions can be done with a two-at-a-time forward stepwise procedure [15]. This forward stepwise selection of basis function leads to an over-fitted model; this means that although it fits the training data well, it becomes a very complex model that is not able to make predictions accurately with new objects. To avoid this issue, basis functions which are redundant are removed one at a time using a backward stepwise procedure. The selection of which basis functions must be included in the model, MARS utilizes generalized cross-validation (GCV). GCV consist of the calculus of the mean squared residual error divided by a penalty dependent on the model complexity. The GCV criterion is defined in the following way:

(4) $$GCV(M)=\frac{\frac{1}{n}{\sum_{i=1}^{n}\left(y_{i}-{\hat{f}}_{M}(\vec{x_{i}})\right)}^{2}}{{\left(1-\frac{C(M)}{n}\right)}^{2}}$$

The model complexity has a penalty, denoted as C(M), that increases with the number of basis functions required by the model, following the next expression:

(5) C(M)=(M+1)+dM

which depends on the number of basis function M, and d is the smoothing parameter that characterizes the penalty for each basis function included into the model. When d takes large values, fewer basis functions are to be included in the model and consequently, smoother function estimates. The selection of the parameter d is discussed in [10]. 

In order to analyse a MARS model, surface plots can be used to visualise the interactions and relations between the basis functions. Let $f_{i}({\vec{x}}_{i})$ be the set of all single variable basis functions that contain only ${\vec{x}}_{i}$. In the same way, $f_{ij}({\vec{x}}_{i},{\vec{x}}_{j})$ is the set of basis functions of two variables, ${\vec{x}}_{i}$ and ${\vec{x}}_{j}$, and $f_{ijk}({\vec{x}}_{i},{\vec{x}}_{j},{\vec{x}}_{k})$ the set of all basis functions of three variables. The MARS model can be rewritten as a series of sums in the following form:

(6) $$\hat{f}(\vec{X})=c_{0}+\sum f_{i}(\vec{x_{i}})+\sum f_{ij}(\vec{x_{i}},\vec{x_{j}})+\sum f_{ijk}(\vec{x_{i}},\vec{x_{j}},\vec{x_{k}})$$

where the first sum is with all the basis functions of one variable, the second is with the basis functions with only two variables. The third sum is over the basis functions of three variables. The expression above is known as ANOVA decomposition since it is similar to the ANOVA decomposition of experimental design [10]. The interaction of a MARS model, based on two variables, is determined by:

(7) $$f_{ij}(\vec{x_{i}},\vec{x_{j}})=f_{i}(\vec{x_{i}})+f_{j}(\vec{x_{j}})+f_{ij}(\vec{x_{i}},\vec{x_{j}})$$

For higher level interactions, they are defined in the same way.

The estimated importance [16,17] of the explanatory variables in the model can be used to construct the basis functions. Determinate predictor importance is, in general, a complex problem that requires several criteria. To obtain reliable results, GCV is usually used to count the number of models subsets (nsubsets) in which each variable is included, and the residual sum of squares (RSS). The definition of the RSS is:

(8) $$RSS=\sum_{i=1}^{n}{\left(y_{i}-{\hat{f}}_{M}(\vec{x_{i}})\right)}^{2}$$

Then, the expression of GCV can be rewritten as:

(9) $$GCV(M)=\frac{RSS/n}{{\left(1-\frac{C(M)}{n}\right)}^{2}}$$

### 2.3. Model Performance Measurement

The performance and accuracy of the trained MARS models can be tested with a comparison of the real efficiency values $y_{i}$ of the validation datasets, and with the values of the predictions ${\hat{y}}_{i}$. The root mean squared error (RMSE), the mean absolute error (MAE) and the R^2^ [18,19] of the model are measures that can be employed with this aim. 

The MAE is a quantity employed to measure how close forecasts are to eventual outcomes [20] and which equation is as follows:

(10) $$MAE=\frac{1}{n}\sum_{i=1}^{n}\left|{\hat{y}}_{i}-y_{i}\right|$$

The RMSE is a general-purpose measure used in a wide range of applications as a measure of the error for numerical predictions [21,22]. Compared to MAE, RMSE amplifies and severely punishes large errors. The RMSE is defined as follows:

(11) $$RMSE=\sqrt{\frac{1}{n}\sum_{i=1}^{n}{\left(y_{i}-{\hat{y}}_{i}\right)}^{2}}$$

### 2.4. Model Training and Validation

The steps followed during the training and validation of all the models are detailed below:

Firstly, data were split in training and validation datasets. As the calculus of efficiency is a regression problem, the function determines the quantiles of the dataset and samples within those groups. The split of the data in training and validation sets was performed using the k-fold cross-validation methodology for k=5. The data was randomly split into five distinct blocks of equal size. Afterwards, the first block of data is left out and the model is fit. This model is used to predict the held-out block. This process was continued until all five held-out blocks had been predicted. A total of 1000 versions of the five-fold cross-validation for both models was created. The k-fold cross-validation is a well-known methodology that was already used in previous studies by the authors with successful results [23,24,25].

For each one of the five models of the five-fold cross-validation, the performance parameters MAE, RMSE, and R^2^ were calculated. This permitted the determination of the minimum, average, and maximum values of those parameters in each one of the 1000 models replications.

After the calculus of all the models referred above, an average model was calculated for the vortex feed scenario and another for the with-media scenario with the two sets of variables, indicating both its performance and the importance of the variables that take part in them.

The 1000 replicas of the five-fold cross-validation methodology are made in order to assure that with independence of the data subset selected, the trained MARS model is able to perform a good prediction of the efficacy. In the case of the average model, it is calculated in order to know the importance of the different variables for the prediction of the efficiency, and also having a parametric model of the LARCODEMS efficiency for both feeds. This model could help in order to know the behaviour of the efficiency variables when changes are performed in both the machine and the volume of flow.

## 3. Results

**Figure 2:**
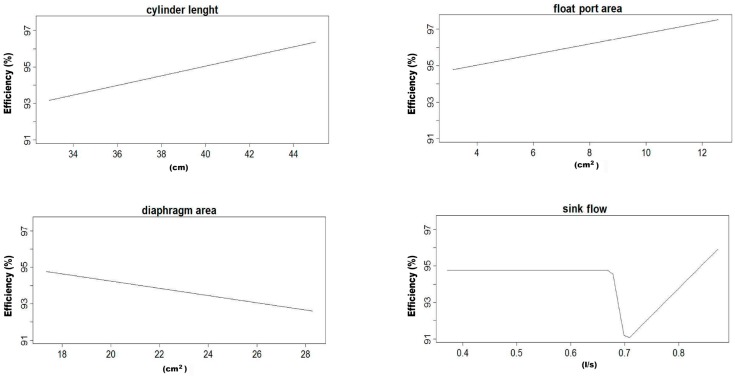
Graphical representation of the basis functions of MARS model number one and their coefficients for feed with media.

**Figure 3:**
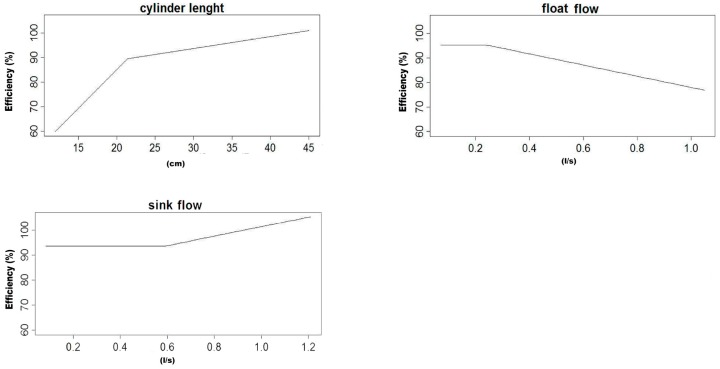
Graphical representation of the basis functions of MARS model number one and their coefficients for feed into the vortex.

**Figure 4:**
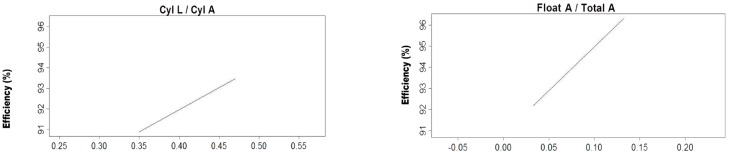
Graphical representation of the basis functions of MARS model number two and their coefficients for feed with media.

**Figure 5:**
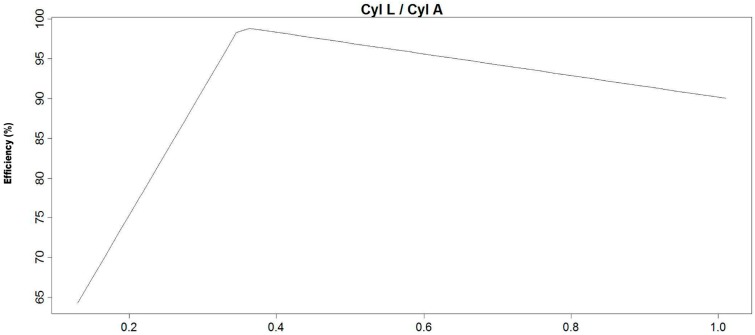
Graphical representation of the basis functions of the MARS model number one and their coefficients for feed into the vortex the ratio CL to CA relative to separation efficiency.

In this work, four average MARS models were obtained for the efficiency of the LARCODEMS device, two with feed into the vortex and another two for feed with media, using the two sets of variables referred in the Materials and Methods section. Their equations are listed in Table 1, Table 2, Table 3 and Table 4. As all the equations are MARS models of the first degree, all their terms can be graphically represented. Please note that the function h(x) is equal to x in when x is larger than 0 and 0 in the rest of the cases. The graphical representation for these models are in Figure 2, Figure 3, Figure 4 and Figure 5, respectively. This helps in the interpretation of the results. Figure 2 corresponds to model number one for feed with the media. It demonstrates that the efficiency of LARCODEMS improves with its length and that there is a linear relation between the improvements in separations obtained and the length of the cylinder. The same relationship also exists between the FPA and efficiency. The increase in efficiency with separation CL can be attributed to the increase in residence time within the separation cylinder and, as such, a greater degree of separation is obtained. Similarly, as the FPA increases, separation media transporting low-density particles increases in thickness, providing more opportunity for only dense particles to pass into the ascending media reporting to the sink port. As the DA increases, efficiency decreases due to the vortex been forced to a diameter where there is insufficient thickness of low-density media resulting in low-density particles reporting to the sink port and, thus, resulting in a decrease in separation efficiency. In general, efficiency is constant with the sink flow, except in a limited number of cases corresponding to data with the minimum CL. Figure 3 shows a substantial increase in efficiency with a cylinder length up to 21.4 cm, and then a less accentuated, but still consistent, increase in efficiency with length up to 44 cm. On the other hand, efficiency is optimum with a minimum FF, and then a maximum after which it consistently decreases with the increase of FF. This decrease in efficiency corresponds to a certain degree of the increase in SF above 0.588 litres per second. Below this value, efficiency is constant. Although in some of the basis functions it would seem possible to reach efficiencies over 100%, as the efficiency in each model is the sum of all the basis functions, none of the MARS models gives an efficiency value above 100% for real working conditions.

Figure 4 shows that, contrary to the results reported by Yang and Wang [8], the separation efficiency is determined by the medium inlet diameter and shape and that the separation density is determined by SP, and efficiency increases as the cylinder length to area increases. Similarly, as the diameter of the float port with respect to the area increases so does the efficiency. In Figure 5 it can be seen how the increases of about 0.09 to 0.35 correspond to an increase in the cylinder length of one of the devices reaching an optimum condition, while the relation of the length to area about 0.35 below this level efficiency tends to decrease.

Results indicated in Figure 4 are dominated by the data for the 110 mm version with 12, 21.4, 32.9, and 45 cm CL and the four diameters of the LARCODEMS versions tested. The increase in separation efficiency for CL/CA values of 0.1–0.38 reflect the increase in the separation efficiency with the increase in CL of the 110 mm model. The subsequent moderate decrease in separation efficiency with an increase in CL/CA from 0.38 to 1.0 is related to the increase in CL/CA with the reduction in the size of the model tested. The efficiency decrease indicates that the separation efficiency obtained increases with the size of the device. This effect is attributed to the increase in the proportion of the surface area affecting the flow of the separation media to the total area of the media going up the separation cylinder, and the relative proportion of thickness of the turbulence layer between the media going up and the media going down, and the separation cylinder to the total area of the separation cylinder occupied by the separation media.

The MARS models make it possible to know which variables take part in each model and their relative importance. Table 5, Table 6, Table 7 and Table 8 indicate the relative importance of the variables. These are presented according to the three metrics for importance classification described in Materials and Method. The classification of these three metrics in all of the models is the same.

**Figure 6:**
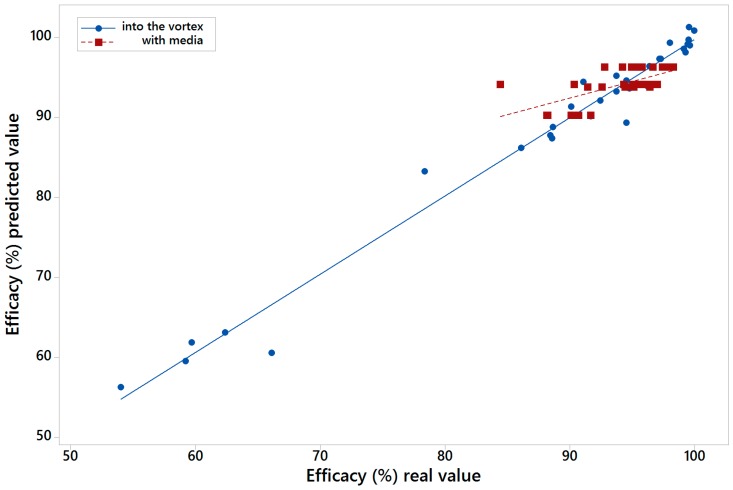
Real value of efficacy versus the calculated by MARS model one for into the vortex and with media feed tests.

**Figure 7:**
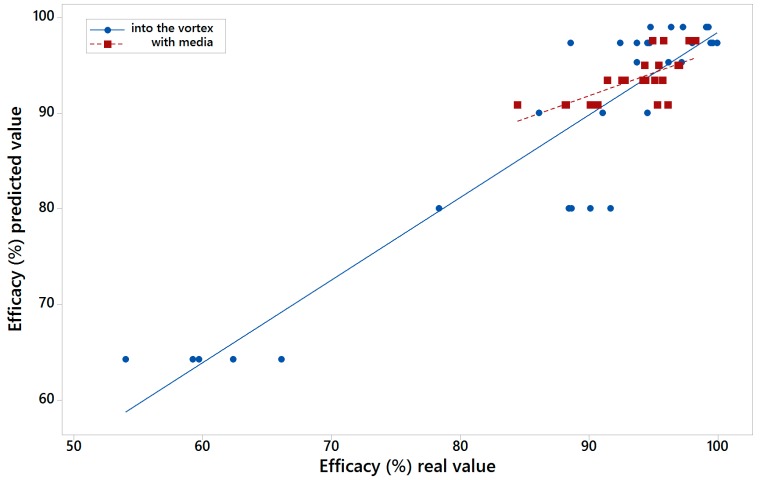
Real value of efficacy versus the calculated by MARS model two for into the vortex and with media feed tests.

Figure 6 shows the real value of efficacy versus the value calculated by MARS model one for into the vortex (blue colour) and with media feed (red colour). Figure 7 shows the same but for those results calculated with the variables of model two. The R^2^ for the models of the LARCODEMS tests performed were as follows: model one with media: 0.6254; into the vortex: 0.9553; model two with media: 0.4820; and into the vortex: 0.8630. This shows a high correlation coefficient of real and predicted efficiencies. In the case of model one, the RMSE, for the test performed with feed into the vortex has a value of 1.9894; while for those test with media feed it is 2.3741. For model two, RMSE values are 2.421 with the media, and 4.8996 into the vortex. In the case of the MAE, for model one with media, the value is 1.7404, and into the vortex, 1.3456. Finally, for model two, the MAE value is 1.8419 with media, and 3.7928 into the vortex. The R^2^ values obtained show a high correlation for the case of the LARCODEMS test performed with media. In the case of the model for the test performed into the vortex, although the correlation is significant, it is not as high. As far as it is known to the authors, there are no other studies that would allow us to compare the RMSE and MAE obtained in this work.

In order to know the performance and stability of those models, the average values of the R^2^, RMSE, and MAE of the 1000 different five-fold cross-validation sets created are summarized in Table 9 for both models one and two with media and into the vortex. The average value of R^2^ is better in the case of both models with into the vortex feed when compared with the model with media feed. In the case of both RMSE and MAE, the average value is larger in the models trained and validated with the into the vortex media test.

## 4. Discussion and Conclusions

In this research, the performance of the MARS models has been tested by means of a five-fold cross-validation methodology. The referred methodology is used to assess the ability of the MARS model to fit to any validation set using, for each model, 5000 different sets (five subsets per test and 1000 replications of the test). The results obtained are in line with previous research that has proven the capability of MARS models to deal with noisy data in the solution, creating a trade-off between the goodness of fit to the amount of data in the structure. Additionally, with the help of MARS models, it is possible to establish an importance order of all of the variables included in the model.

From a technical perspective, it is shown that:

- Separation efficiencies increase with both the length and size of the separation cylinder. This is reflected by the relation between the separation efficiency and media flow rates in the sink and float ports and by the relation between the cylinder length to the cylinder area.
- For a given separation cylinder size there is an optimum length above which the separation efficiency does not increase.

The results of this investigation indicate that the LARCODEMS is a relatively robust density separation device suitable for obtaining efficient separations, provided that a suitable CL is maintained. It is indicated that since the FF and SF rates are related to the SPA, FPA, and the DA, such separations may be optimized based on an adequate control of the FPA and, to a lesser extent, the DA.

The increase in separation efficiency with a decrease in CL/CA indicates that, for this type of DMS cyclone, the larger it is, the better the results. Furthermore, unlike the cono-cylindrical type of DMS, the diameter of the cyclone does not appear to be limited to the >500 micron particle size that may be treated, and supports the observations of previous works [6,7].

It is suggested that this methodology could also be applied to analyse the operation of other heavy media separator density separations to optimize the efficiencies obtained. Finally, we would like to also remark that, as far as it is known by the authors, the efficiency model proposed in this research is the first parametric model proposed for the efficiency of a LARCODEMS device.

## Figures and Tables

**Table 1:** List of basis functions of multivariate adaptive regression splines (MARS) model number one and their coefficients for feed with media.

B_i_	Definition	C_i_
B_1_	Constant	87.045425
B_2_	Cylinder length	0.263318
B_3_	Float port area	0.292959
B_4_	Diaphragm area	–0.198685
B_5_	h(Sink flow − 0.677)	–164.231069
B_6_	h(Sink flow − 0.677)	193.832769

**Table 2:** List of basis functions of MARS model number one and their coefficients for feed into the vortex.

B_i_	Definition	C_i_
B_1_	Constant	88.024967
B_2_	h(21.4 − Cylinder length)	–3.149586
B_3_	h(Cylinder length − 21.4)	0.491264
B_4_	h(Float flow − 0.243)	–22.817374
B_5_	h(Sink flow − 0.588)	19.200122

**Table 3:** List of basis functions of MARS model number two and their coefficients for feed with media.

B_i_	Definition	C_i_
B_1_	Constant	81.97549
B_2_	Cylinder length/Cylinder Area	21.50694
B_3_	Float port area/Total Area	41.73611

**Table 4:** List of basis functions of the MARS model number two and their coefficients for feed into the vortex.

B_i_	Definition	C_i_
B_1_	constant	99.00408
B_2_	h(0.35 − Cylinder length/Cylinder Area)	–157.73737
B_3_	h(Cylinder length/Cylinder Area − 0.35)	–13.58808

**Table 5:** Relative importance of variables in MARS model number one and their coefficients for feed with media.

Variable	Nsubsets	GCV	RSS
Diaphragm area	5	100	100
Cylinder length	4	69.9	79.3
Sink flow	3	41.8	61.7
Float port area	1	13.2	32.3

**Table 6:** Relative importance of variables in MARS model number one and their coefficients for feed into the vortex.

Variable	Nsubsets	GCV	RSS
Cylinder length	4	100	100
Float flow	1	17.2	16.4
Sink flow	1	17.2	16.4

**Table 7:** List of basis functions of MARS model number two and their coefficients for feed with media.

Variable	Nsubsets	GCV	RSS
Float A/Total A	2	100	100
Cyl L/Cyl A	1	40.1	55.2

**Table 8:** List of basis functions of MARS model number two and their coefficients for feed into the vortex.

Variable	Nsubsets	GCV	RSS
Cyl L/Cyl A	2	100	100

**Table 9:** Performance measurements of models one and two trained with the media feed and the feed into the vortex.

Variable	With Media			Into the Vortex		
MODEL 1	Min.	Avg.	Max.	Min.	Avg.	Max.
*R*^2^	0.3543	0.5080	0.6776	0.6303	0.7001	0.8078
RMSE	2.1672	2.9757	6.3582	7.4346	9.2680	22.1758
MAE	1.8560	2.3265	4.8040	6.2205	7.9052	15.6619
MODEL 2	Min.	Avg.	Max.	Min.	Avg.	Max.
*R*^2^	0.3355	0.4333	0.5519	0.6838	0.7785	0.8601
RMSE	2.6046	3.3521	4.6182	5.5193	7.4982	13.0074
MAE	2.1771	2.7911	4.0124	4.3370	6.2126	10.9078
